# Risk of a permanent work-related disability pension after incident venous thromboembolism in Denmark: A population-based cohort study

**DOI:** 10.1371/journal.pmed.1003770

**Published:** 2021-08-31

**Authors:** Helle Jørgensen, Erzsébet Horváth-Puhó, Kristina Laugesen, Sigrid Brækkan, John-Bjarne Hansen, Henrik Toft Sørensen

**Affiliations:** 1 Thrombosis Research Center (TREC), Department of Clinical Medicine, UiT, The Arctic University of Norway, Tromsø, Norway; 2 Department of Clinical Epidemiology, Aarhus University Hospital, Aarhus, Denmark; 3 Division of Internal Medicine, University Hospital of North Norway, Tromsø, Norway; Stanford University, UNITED STATES

## Abstract

**Background:**

Long-term complications of venous thromboembolism (VTE) hamper physical function and impair quality of life; still, it remains unclear whether VTE is associated with risk of permanent work-related disability. We aimed to assess the association between VTE and the risk of receiving a permanent work-related disability pension and to assess whether this association was explained by comorbidities such as cancer and arterial cardiovascular disease.

**Methods and findings:**

A Danish nationwide population-based cohort study consisting of 43,769 individuals aged 25 to 66 years with incident VTE during 1995 to 2016 and 218,845 birth year-, sex-, and calendar year-matched individuals from the general population, among whom 45.9% (*N =* 120,540) were women, was established using Danish national registries. The cohorts were followed throughout 2016, with permanent work-related disability pension as the outcome. Hazard ratios (HRs) with 95% confidence intervals (CIs) for disability pension were computed and stratified by sex and age groups (25 to 34, 35 to 44, 45 to 54, and 55 to 66 years of age) and adjusted for comorbidities and socioeconomic variables.

Permanent work-related disability pensions were granted to 4,415 individuals with VTE and 9,237 comparison cohort members (incidence rates = 17.8 and 6.2 per 1,000 person-years, respectively). VTE was associated with a 3-fold (HR 3.0, 95% CI: 2.8 to 3.1) higher risk of receiving a disability pension. Adjustments for socioeconomic status and comorbidities such as cancer and cardiovascular diseases reduced the estimate (HR 2.3, 95% CI: 2.2 to 2.4). The risk of disability pension receipt was slightly higher in men than in women (HR 2.5, 95% CI: 2.3 to 2.6 versus HR 2.1, 95% CI: 2.0 to 2.3). As this study is based on medical and administrative registers, information on post-VTE care, individual health behavior, and workplace factors linked to disability pension in the general population are lacking. Furthermore, as disability pension schemes vary, our results might not be directly generalizable to other countries or time periods.

**Conclusions:**

In this study, incident VTE was associated with increased risk of subsequent permanent work-related disability, and this association was still observed after accounting for comorbidities such as cancer and cardiovascular diseases. Our results emphasize the social consequences of VTE and may help occupational and healthcare professionals to identify vulnerable individuals at risk of permanent exclusion from the labor market after a VTE event.

## Abstract 2

In a population-based cohort study, Helle Jørgensen and colleagues investigate the risk of receiving a permanent work-related disability pension after incident venous thromboembolism in Denmark.

## Author summary

**Why was this study done?:**

- Long-term complications of venous thromboembolism (VTE) are known to hamper functional activities and impair quality of life.
- Existing research on the risk of permanent exclusion from the labor marked after a VTE is scarce.

**What did the researchers do and find?:**

- In this large nationwide study of >250,000 individuals, we found that VTE is associated with a 3-fold (hazard ratio (HR) 3.0, 95% confidence interval (CI): 2.8 to 3.1) higher risk of permanent work-related disability compared to the general population.
- The association between VTE and disability pension was still observed (HR 2.3, 95% CI: 2.2 to 2.4) after adjustments for socioeconomic status and comorbidities such as cancer and cardiovascular diseases.

**What do these findings mean?:**

- Patients suffering from a VTE are at risk of permanent exclusion from the labor market.
- Our findings emphasize the social consequences of VTE and can contribute to further improvement of protective strategies to diminish the burden of VTE on public health and healthcare systems.
- As disability pension schemes vary, our results might not be directly generalizable to other countries or time periods.
- Even though we have adjusted for multiple comorbidities and socioeconomic factors, residual confounding (e.g., due to individual health behavior or work place factors) cannot be completely ruled out.

## Introduction

Venous thromboembolism (VTE), encompassing deep vein thrombosis (DVT) and pulmonary embolism (PE), is a prevalent multifactorial disease with an incidence rate (IR) of 1 to 2 per 1,000 person-years in adults [1,2]. Despite increased public awareness of the disease and availability of preventive measures, VTE incidence has increased during the past decades [3]. Severe complications such as increased mortality risk [4], recurrence [5,6], post-thrombotic syndrome (PTS) [7,8], and post-pulmonary embolism (post-PE) syndrome [9–13] may cause reduced mobility, lessened capacity for work, and a lowered quality of life (QoL) in a large proportion of individuals with VTE [7,8,14–17].

Although VTE has been documented as a leading cause of lost disability-adjusted life-years, existing research on work-related disability and socioeconomic consequences following a VTE is scarce [5,17]. Two European multicenter studies including 1,399 individuals with PE and 2,056 individuals with DVT found that 27.8% of those with PE and 29.5% of those with DVT had not returned to work 1 year after their VTE diagnosis [18,19]. However, these studies did not adjust for other comorbidities, and the follow-up time was limited to 12 months [18,19]. A Norwegian cohort study of 66,005 individuals, including 386 individuals with VTE, with 14 years of follow-up, reported a 37% increased risk of permanent work-related disability in individuals with VTE compared to the general population, with a higher risk in individuals with DVT than in individuals with PE [14]. Thus, VTE represents a major burden to public health and healthcare systems [16,17,20]. Further research on the work-related consequences of VTE is required to improve protective strategies that diminish the indirect costs and social burden of the disease. The aim of this nationwide population-based cohort study was to assess the risk of receiving a permanent work-related disability pension among patients with VTE compared to individuals from the general population without VTE, according to age, sex, and VTE subtypes. As VTE often occurs secondary to comorbidities such as cancer or cardiovascular disease (i.e., myocardial infarction and stroke) [2,21,22], we also investigated whether a potential association between VTE and receipt of a permanent disability pension was explained by comorbidities.

## Methods

### Design and setting

The prospective analysis plan for this study can be found in the supporting information (S1 Analysis plan). This study is reported as per the Strengthening the Reporting of Observational Studies in Epidemiology (STROBE) guideline (S1 Checklist).

We conducted this population-based cohort study using linked data from Danish administrative and medical databases. The Danish healthcare system is government funded, ensuring free and equal access to tax-supported healthcare for all legal residents [23]. Around 90% of Denmark’s population is of Danish descent, defined as having at least 1 parent who was born in Denmark and has Danish citizenship. Upon birth or immigration, the Danish Civil Registration System (CRS) assigns a unique 10-digit personal identifier (CPR number) to all Danish residents. The CPR number allows accurate and complete individual-level linkage among all Danish registries, as well as tracking of study participants over time, allowing accurate censoring due to emigration or death [23].

### VTE cohort

The Danish National Patient Registry (DNPR) contains data on all nonpsychiatric discharges from Danish hospitals since 1977 and on psychiatric inpatients, emergency department, and outpatient specialty clinic contacts since 1995 [24]. DNPR data permit individual-level identification of patients’ medical histories. Using ICD-10 codes, we searched the DNPR to identify all inpatients and outpatients with a primary or secondary diagnosis of DVT or PE in the period January 1, 1995 through December 31, 2016. The first hospital admission/outpatient visit date defined the VTE date. We excluded individuals already receiving a work-related disability pension and patients with a VTE diagnosis before 1994 (identified using ICD-8 codes). Patients aged <25 years and patients retired from work due to advanced age at study inclusion (>66 years) were also excluded, as they were unlikely to be eligible for the outcome. VTE registered solely in emergency room departments were excluded, as they frequently represent working diagnoses with high rates of clinical misclassification [25].

We classified VTE as either DVT or PE. If a patient had a simultaneous PE and DVT diagnosis, we used the PE diagnosis due to its higher mortality rate [4]. In addition, VTEs accompanied by a preexisting cancer diagnosis whenever before or on index date, in addition to fracture, trauma, surgery, and/or pregnancy within 90 days prior to VTE diagnosis, were classified as provoked VTE, while VTEs in the absence of these factors were classified as unprovoked VTE [26].

### General population comparison cohort

We used the CRS to sample a population-based comparison cohort. For each VTE patient, up to 5 individuals from the general population were randomly matched on sex, year of birth, and calendar year, with replacement [27]. A VTE patient’s hospital admission date was defined as the index date for the comparison cohort members. Individuals in the comparison cohort could not have been hospitalized for VTE or have received a permanent work-related disability pension prior to the index date. If a person from the comparison cohort subsequently experienced a VTE, he/she was censored and moved to the exposure cohort from that date onwards.

### Outcome

We extracted information on receipt of a work-related disability pension from the Integrated Database for Labour Market Research (IDA) at Statistics Denmark. This database has covered the employment status, workplace, and employers of the entire Danish population yearly since 1980 [28]. Danish residents are entitled to a disability pension if their capacity for work is substantially and permanently reduced to such a degree that they will never be able to provide for themselves through regular or flexible work [29]. All persons who have permanent legal residence in Denmark and who have lived in Denmark for at least 3 years since their 15th birthday are eligible for a disability pension until public retirement age [29]. A 2013 legislative reform made granting of a disability pension to persons younger than age 40 years much stricter [29].

A direct transition from work to receipt of a disability pension rarely occurs, as most persons go through a period of sick leave and measures to improve their capacity for work before a disability pension is granted [30]. We therefore excluded individuals with VTE and comparison cohort members who had received a disability pension in the same or previous calendar year as their VTE/index date. This way we avoided potential bias and ensured that the VTE itself was the reason for disability, rather than a consequence of another condition. Receipt of a disability pension was ascertained annually at the end of November of a given calendar year, and we defined the disability pension date as January 1 of that year.

It is important to note that disability can vary in both length and severity and that it does not necessarily lead to a permanent exit from work life. Measuring permanent receipt of a disability pension as our study outcome might therefore underestimate the actual incidence of disability caused by VTE in the general population.

### Cohort characteristics

We obtained information on comorbidities diagnosed prior to the VTE/index date using ICD-8 and ICD-10 codes from the DNPR for obesity, cancer, coronary heart disease (including atrial fibrillation), diabetes, stroke, chronic obstructive pulmonary disease (COPD), acute kidney failure and chronic kidney disease, surgery 3 months prior to the VTE/index date, and diseases included in the Charlson Comorbidity Index [31]. All ICD codes used in the study are provided in S4 Table.

We measured socioeconomic status (SES) based on education, employment status, and income. Information on education was obtained from the Educational Attainment Register for the year prior to the VTE/index date. We divided the level of education (i.e., low, medium, and high) into age-specific groups based on the distribution of education in each group (S2 and S3 Tables). Income and employment status were extracted from the IDA. To avoid the impact of inflation and to account for salary changes over calendar time, we calculated yearly income quartiles for each index year based on the previous year (e.g., quartiles in 2001 were calculated based on the income of exposed and unexposed persons with an index date in 2000). The 2 middle quartiles were merged to yield 3 categories (i.e., low, medium, and high). Employment status was measured the year prior to VTE/index date and categorized as “employed, unemployed, and outside the workforce.” The category “outside the workforce” included persons in an educational program, those in early retirement, and those receiving other types of public support. Employment status was categorized as low (i.e., unemployed), medium (i.e., outside the workforce), and high (i.e., employed).

Using the “low, medium, and high” categorical distributions described above, we created an SES index for education, income, and employment status ranging from 1 to 3 for each category. The total SES index score thus ranged from 3 to 9. Based on the distribution of the total index sum score, we divided the SES index into high (i.e., scores of 8 and 9), medium (i.e., scores of 5 to 7), and low (i.e., scores of 3 and 4) SES, with high SES serving as reference.

### Statistical analysis

We followed all cohort members from their index date until January 1 the year the work-related disability pension was recorded, emigration from Denmark, date of death, age 66, or end of the study period (December 31, 2016), whichever came first. Persons who turned 66 years old, emigrated, or died during follow-up were censored on the date of the event. Crude IRs were calculated as number of events per 1,000 person-years at risk. We used stratified Cox proportional hazards regression models to compute unadjusted and adjusted hazard ratios (HRs) as a measure of work-related disability with 95% confidence intervals (CIs). The proportional hazards assumption was tested using log–log plots and found not violated. Additionally, we explored sensitivities around thresholds of different ages against a confounder-adjusted restricted cubic spline with a prespecified list of 5 knots with age = 30 as reference (S1 Fig) and performed age- and sex-stratified analyses (age groups: 25 to 34 years, 35 to 44 years 45 to 54 years, and 55 to 66 years at index date).

We adjusted the HRs for a priori-defined potential confounding, including comorbidities, using 3 different models stratified by age group and sex. Model 1 was unadjusted and controlled for matching variables by study design. Model 2 additionally included SES index and obesity; Model 3 included adjustment for SES index score, obesity, cancer, coronary heart disease (including atrial fibrillation), diabetes, stroke, COPD, acute kidney failure/chronic kidney disease, and surgery 3 months prior to the VTE/index date, and the Charlson Comorbidity Index score (excluding comorbidities already adjusted for). We also performed age- and sex-stratified subgroup analyses with PE, DVT, and unprovoked and provoked VTE as exposure variables.

As the mortality rates were likely to differ among those with and without VTE, the rates of disability pension could potentially be overestimated as a result of competing risk of death. In order to account for death as a competing event, cumulative incidence functions were estimated by the methods proposed by Fine and Gray [32] and visualized according to VTE/no VTE and age groups (age 25 to 34 years, 35 to 44 years, 45 to 54 years, and 55 to 66 years).

All analyses were conducted using SAS version 9.4 (SAS Institute, Cary, NC).

### Ethics

According to Danish legislation, registry-based studies do not require informed consent and approval from an ethics committee. The study was approved by the Danish Data Protection Agency (record no.2016-051-000001).

## Results

We identified 43,769 individuals with VTE and 218,845 matched individuals from the general population aged 25 to 66 years at inclusion, among whom 45.9% (*N =* 120,540) were women. Baseline characteristics for individuals with VTE and members of the comparison cohort are provided in Table 1. VTE was associated with a higher proportion of individuals with low or medium SES than the comparison cohort (62.1% versus 56.8%), and more comorbidity, such as surgery 3 months prior to VTE/index date (14.6% versus 2.5%), history of cancer (12.2% versus 3.2%), coronary heart disease (including atrial fibrillation) (6.7% versus 4.2%), COPD (5.8% versus 3.0%), obesity (6.1% versus 2.6%), and diabetes (5.5% versus 3.8%) (Table 1).

**Table 1:** Baseline characteristics of persons with VTE and members of the general comparison cohort.

	VTE (*n =* 43,769)	Comparison cohort (*n =* 218,845)
**Sex (% women)**	20,090 (45.9)	100,450 (45.9)
**PE**	15,006 (34.3)	
**DVT**	28,763 (65.7)	
**Unprovoked VTE**	32,788 (74.9)	
**Provoked VTE**	10,981 (25.1)	
**SES score**		
Low	5,173 (11.8)	18,334 (8.4)
Medium	22,037 (50.3)	105,921 (48.4)
High	15,308 (35.0)	88,533 (40.5)
Missing	1,251 (2.9)	6,057 (2.8)
**Comorbidities**		
Cancer	5,347 (12.2)	7,108 (3.2)
Coronary heart disease	2,948 (6.7)	9,201 (4.2)
Diabetes	2,406 (5.5)	8,321 (3.8)
COPD	2,537 (5.8)	6,531 (3.0)
Obesity	2,675 (6.1)	5,693 (2.6)
Stroke	772 (1.8)	2,054 (0.9)
Moderate to severe renal disease	767 (1.8)	1,221 (0.6)
Surgery 3 months prior to VTE/index date	6,410 (14.6)	5,364 (2.5)
Pregnancy 3 months prior VTE/index date	667 (1.5)	1,029 (0.5)
Trauma/fracture 3 months prior to VTE/index date	1,904 (4.4)	1,299 (0.6)
CCI*		
CCI score: 0	38,488 (87.9)	206,732 (94.5)
CCI score: 1	4,346 (9.9)	10,660 (4.9)
CCI score: > = 2	935 (2.1)	1,453 (0.7)

*CCI: Modified Charlson Comorbidity Index excluding ICD codes used in the covariate definition.

Values are numbers, with percentages in brackets.

COPD, chronic obstructive pulmonary disease; DVT, deep vein thrombosis; PE, pulmonary embolism; SES, socioeconomic status; VTE, venous thromboembolism.

Permanent work-related disability pensions were granted to 13,652 persons (5.2%) during a median overall follow-up time of 4.9 years (Table 2). Among pension recipients, 4,415 were individuals with VTE (10.1% of all individuals with VTE) (Table 2). Individuals with VTE who subsequently received a disability pension were characterized by a higher proportion of women (48.4% versus 45.6%) and a higher proportion of individuals with DVT (69.4% versus 65.3%) than individuals with VTE without disability pension (S2 Table).

**Table 2:** IRs and HRs with 95% CIs by age for receipt of a work-related DP among persons with and without VTE.

	Comparison cohort			VTE					
	No DP	DP	IR (95% CI)	No DP	DP	IR (95% CI)	Model 1 HR (95% CI)	Model 2 HR (95% CI)	Model 3 HR (95% CI)
Overall	218,845	9,237	6.2 (6.0–6.3)	43,769	4,415	17.8 (17.3–18.3)	3.0 (2.8–3.1)	2.7 (2.6–2.8)	2.3 (2.2–2.4)
Age 25–34	27,072	825	3.1 (2.9–3.3)	5,393	550	11.5 (10.5–12.4)	3.9 (3.5–4.4)	3.0 (2.7–3.4)	2.7 (2.4–3.1)
Age 35–44	42,046	2,049	5.2 (5.0–5.4)	8,412	1,091	16.5 (15.5–17.5)	3.3 (3.0–3.5)	2.8 (2.5–3.0)	2.5 (2.2–2.7)
Age 45–54	59,765	3,610	7.4 (7.1–7.6)	11,962	1,623	20.6 (19.6–21.6)	2.7 (2.6–2.9)	2.6 (2.4–2.8)	2.1 (2.0–2.3)
Age 55–66	89,962	2,753	7.9 (7.6–8.2)	18,002	1,151	20.9 (19.7–22.1)	2.7 (2.5–2.9)	2.7 (2.5–2.9)	2.2 (2.0–2.4)

Model 1: unadjusted model controlled for matching variables by study design.

Model 2: adjusted for SES score (education, employment status, and income) and obesity.

Model 3: adjusted for SES score (education, employment status, and income), obesity, cancer, coronary heart disease (including atrial fibrillation), diabetes, stroke, COPD, acute kidney failure and chronic kidney disease, surgery 3 months prior to the VTE/index date, and Charlson Comorbidity Index score, excluding comorbidities already adjusted for.

CI, confidence interval; COPD, chronic obstructive pulmonary disease; DP, disability pension; HR, hazard ratio; IR, incidence rate; SES, socioeconomic status; VTE, venous thromboembolism.

The IR of disability in individuals with VTE was 17.8 (95% CI: 17.3 to 18.3) compared to 6.2 (95% CI: 6.0 to 6.3) in the comparison cohort (Table 2). This corresponded to an absolute rate difference for work-related disability pension in individuals with and without incident VTE of 11.6 events per 1,000 person-years at risk. In the unadjusted model, incident VTE was associated with a 3-fold (HR 3.0, 95% CI: 2.8 to 3.1) increased risk of subsequently receiving a disability pension (Table 2). The risk estimate for receipt of a disability pension decreased to 2.7-fold (HR 2.7, 95% CI: 2.6 to 2.8) after adjusting for the SES index score and obesity. Further adjustment for comorbidities (Model 3) attenuated the risk estimate (HR 2.3, 95% CI: 2.2 to 2.4) (Table 2).

Although the IR of disability increased with age for both individuals with VTE and the general population comparison group, we found that the HR for subsequent receipt of a disability pension decreased with increasing age (Table 2). After adjusting for SES and comorbidities, the HR for receiving a disability pension among individuals with VTE decreased from 2.7 (95% CI: 2.4 to 3.1) in the youngest age group to HR 2.2 (95% CI: 2.0 to 2.4) in the oldest age group (Model 3). Of note, there was an increase in the absolute risk difference from 8.4 per 1,000 person-years in the youngest age group to 13.0 per 1,000 person-years in the oldest age group (Table 2).

The IR of disability in the general comparison cohort was higher overall for women (IR 6.7, 95% CI: 6.5 to 6.9) than for men (IR 5.6, 95% CI: 5.5 to 5.8). However, VTE was associated with a somewhat higher IR for disability in men than in women (IR 18.1, 95% CI: 17.3 to 18.8, versus IR 17.5, 95% CI: 16.8 to 18.3) (Table 3). VTE remained associated with a higher relative risk of receiving a disability pension in men (HR 2.5, 95% CI: 2.3 to 2.6) than in women (HR 2.1, 95% CI: 2.0 to 2.3), even after adjusting for SES and comorbidities.

**Table 3:** IRs and HRs with 95% CIs by sex and age of receipt of a work-related DP for persons with and without VTE.

	Comparison cohort			VTE					
							Model 1	Model 2	Model 3
	No DP	DP	IR (95% CI)	No DP	DP	IR (95% CI)	HR (95% CI)	HR (95% CI)	HR (95% CI)
**MEN**									
Overall	118,395	4,335	5.6 (5.5–5.8)	23,679	2,279	18.1 (17.3–18.8)	3.2 (3.0–3.4)	2.9 (2.7–3.1)	2.5 (2.3–2.6)
Age 25–34	9,050	283	3.1 (2.7–3.4)	1,804	243	15.6 (13.6–17.5)	5.3 (4.4–6.3)	3.5 (2.8–4.4)	3.2 (2.5–4.0)
Age 35–44	19,618	885	4.7 (4.4–5.1)	3,923	551	18.0 (16.5–19.5)	4.0 (3.6–4.5)	3.2 (2.8–3.7)	2.9 (2.5–3.4)
Age 45–54	33,841	1,810	6.6 (6.3–6.9)	6,757	876	19.6 (18.3–20.9)	2.9 (2.6–3.1)	2.7 (2.5–3.0)	2.3 (2.0–2.5)
Age 55–66	55,886	1,357	6.3 (6.0–6.6)	11,195	609	17.2 (15.9–18.6)	2.8 (2.5–3.1)	2.8 (2.5–3.1)	2.2 (2.0–2.5)
**WOMEN**									
Overall	100,450	4,902	6.7 (6.5–6.9)	20,090	2,136	17.5 (16.8–18.3)	2.7 (2.6–2.9)	2.5 (2.3–2.6)	2.1 (2.0–2.3)
Age 25–34	18,022	542	3.1 (2.8–3.3)	3,589	307	9.5 (8.4–10.5)	3.2 (2.8–3.7)	2.7 (2.3–3.2)	2.5 (2.1–2.9)
Age 35–44	22,428	1,164	5.6 (5.3–6.0)	4,489	540	15.2 (13.9–16.5)	2.7 (2.5–3.1)	2.4 (2.1–2.7)	2.1 (1.9–2.4)
Age 45–54	25,924	1,800	8.3 (7.9–8.7)	5,205	747	21.9 (20.3–23.5)	2.6 (2.4–2.8)	2.4 (2.2–2.6)	2.0 (1.8–2.2)
Age 55–66	34,076	1,396	10.7 (10.1–11.2)	6,807	542	27.3 (25.0–29.6)	2.6 (2.4–2.9)	2.6 (2.3–2.9)	2.2 (1.9–2.4)

Model 1: unadjusted model controlled for matching variables by study design.

Model 2: adjusted for SES (education, employment status, and income) and obesity.

Model 3: adjusted for SES score (education, employment status, and income), obesity, cancer, coronary heart disease (including atrial fibrillation), diabetes, stroke, COPD, acute kidney failure and chronic kidney disease, surgery 3 months prior to the VTE/index date, and Charlson Comorbidity Index score, excluding comorbidities already adjusted for.

CI, confidence interval; COPD, chronic obstructive pulmonary disease; DP, disability pension; HR, hazard ratio; IR, incidence rate; SES, socioeconomic status; VTE, venous thromboembolism.

The sex-specific difference in relative risk was largest in the youngest age group. VTE was associated with a 3.2-fold higher risk of disability pension in men (HR 3.2, 95% CI: 2.5 to 4.0) and 2.5-fold higher risk in women (HR 2.5, 95% CI: 2.1 to 2.9) aged 25 to 34 years, compared to the general population (Table 3). The HR for receiving a disability pension in men decreased gradually with increasing age from 3.2 (95% CI 2.5 to 4.0) in the age group 25 to 34 years to 2.2 (95% CI: 2.0 to 2.5) in the age group 55 to 66 years. Only a modest decline in the HR among women with VTE was observed across age groups. The absolute rate difference in disability pension receipt among men remained relatively stable at 13 per 1,000 person-years, while the absolute rate difference in women increased from 6.4 per 1,000 person-years in the age group 25 to 34 years to 16.6 per 1,000 person-years in the age group 55 to 66 years.

PE was associated with a higher relative risk of receiving a disability pension than DVT (Table 4). PE was associated with a 2.6-fold (HR 2.6, 95% CI: 2.4 to 2.8) higher risk and DVT a 2.2-fold higher risk (HR 2.2, 95% CI: 2.0 to 2.3) of receiving a disability pension compared to persons without VTE, after adjusting for SES and comorbidities (Table 4). For PE, the overall risk of receiving a disability pension was similar in men (HR 2.7, 95% CI: 2.4 to 3.0) and in women (HR 2.6, 95% CI: 2.3 to 2.9), while DVT showed a higher risk estimate for receipt of a disability pension in men (HR 2.4, 95% CI: 2.2 to 2.6) than in women (HR 2.0, 95% CI: 1.8 to 2.1).

**Table 4:** Subgroup analysis by age with IRs and HRs with 95% CIs by age for receipt of a work-related DP for persons with and without VTE (PE vs. DVT).

		Comparison cohort			VTE		Model 1	Model 2	Model 3
	No DP	DP	IR (95% CI)	No DP	DP	IR (95% CI)	HR (95% CI)	HR (95% CI)	HR (95% CI)
**PE**									
Overall	75,030	2,836	6.3 (6.0–6.5)	15,006	1,351	19.5 (18.4–20.5)	3.2 (3.0–3.5)	3.1 (2.9–3.4)	2.6 (2.4–2.8)
Age 25–34	7,927	228	3.2 (2.8–3.6)	1,575	128	10.1 (8.4–11.9)	3.5 (2.8–4.3)	2.8 (2.2–3.6)	2.6 (2.0–3.4)
Age 35–44	12,514	560	5.2 (4.7–5.6)	2,515	275	15.4 (13.5–17.2)	3.0 (2.6–3.5)	2.9 (2.4–3.4)	2.6 (2.1–3.1)
Age 45–54	19,315	1,058	7.2 (6.8–7.6)	3,852	496	23.2 (21.1–25.2)	3.2 (2.8–3.5)	3.0 (2.7–3.5)	2.5 (2.2–2.9)
Age 55–66	35,274	990	7.9 (7.4–8.4)	7,064	452	25.9 (23.5–28.3)	3.4 (3.0–3.8)	3.5 (3.0–3.9)	2.8 (2.4–3.2)
**DVT**									
Overall	143,815	6,401	6.1 (6.0–6.3)	28,763	3,064	17.2 (16.5–17.8)	2.8 (2.7–3.0)	2.5 (2.4–2.6)	2.2 (2.0–2.3)
Age 25–34	19,145	597	3.0 (2.8–3.3)	3,818	422	11.9 (10.8–13.1)	4.1 (3.6–4.6)	3.1 (2.7–3.6)	2.8 (2.3–3.2)
Age 35–44	29,532	1,489	5.2 (5.0–5.5)	5,897	816	17.0 (15.8–18.1)	3.4 (3.1–3.7)	2.7 (2.4–3.0)	2.4 (2.1–2.7)
Age 45–54	40,450	2,552	7.4 (7.1–7.7)	8,110	1,127	19.6 (18.5–20.8)	2.6 (2.4–2.8)	2.4 (2.2–2.6)	2.0 (1.8–2.2)
Age 55–66	54,688	1,763	8.0 (7.6–8.3)	10,938	699	18.5 (17.2–19.9)	2.4 (2.2–2.6)	2.3 (2.1–2.5)	1.9 (1.7–2.1)

Model 1: unadjusted model controlled for matching variables by study design.

Model 2: adjusted for SES (education, employment status, and income) and obesity.

Model 3: adjusted for SES score (education, employment status, and income), obesity, cancer, coronary heart disease (including atrial fibrillation), diabetes, stroke, COPD, acute kidney failure and chronic kidney disease, surgery 3 months prior to the VTE/index date, and Charlson Comorbidity Index score, excluding comorbidities already adjusted for.

CI, confidence interval; COPD, chronic obstructive pulmonary disease; DP, disability pension; DVT, deep vein thrombosis; HR, hazard ratio; IR, incidence rate; PE, pulmonary embolism; SES, socioeconomic status; VTE, venous thromboembolism.

The overall risk of receiving a disability pension following a PE remained relatively stable across age groups, while the risk after a DVT decreased with advancing age (Table 5). VTE in men in the age group 25 to 34 years was associated with a 3.1-fold higher risk of subsequent receipt of disability pension after both PE (HR 3.1, 95% CI: 1.8 to 5.2) and DVT (HR 3.2, 95% CI: 2.5 to 4.2), after adjustment for SES and comorbidities (Model 3). These risk estimates decreased to HR 2.8 for PE (95% CI: 2.3 to 3.4) and HR 1.9 for DVT (95% CI: 1.7 to 2.3) in the age group 55 to 66 years (Table 5). In women in the age group 25 to 34 years, PE was associated with a 2.4-fold (HR 2.4, 95% CI: 1.7 to 3.3) higher risk of subsequent receipt of a disability pension. The corresponding HR in women in the age group 55 to 66 years was 2.8 (95% CI: 2.3 to 3.4). The risk of receiving a disability pension in women with DVT decreased gradually, compared to women without VTE. The association decreased from 2.5-fold (HR 2.5, 95% CI: 2.0 to 3.0) in the age group 25 to 34 years to 1.9-fold (HR 1.9, 95% CI: 1.6 to 2.2) in the age group 55 to 66 years (Table 5).

**Table 5:** Subgroup analysis by age and sex with IRs and HRs with 95% CIs of receipt of a work-related DP among persons with and without VTE (PE versus DVT).

	Comparison cohort		VTE		Model 1	Model 3
	No DP	DP	No DP	DP	HR (95% CI)	HR (95% CI)
**PE**						
PE Men overall	40,270	1,331	8,054	696	3.4 (3.1–3.7)	2.7 (2.4–3.0)
PE Men age 25–34	2,321	63	459	50	5.2 (3.5–7.9)	3.1 (1.8–5.2)
PE Men age 35–44	5,589	245	1,124	123	3.1 (2.4–3.9)	2.6 (1.9–3.4)
PE Men age 45–54	10,833	526	2,156	273	3.3 (2.8–3.8)	2.6 (2.1–3.1)
PE Men age 55–66	21,527	497	4,315	250	3.4 (2.9–4.0)	2.8 (2.3–3.4)
PE Women overall	34,760	1,505	6,952	655	3.1 (2.8–3.4)	2.6 (2.3–2.9)
PE Women age 25–34	5,606	165	1,116	78	2.8 (2.2–3.8)	2.4 (1.7–3.3)
PE Women age 35–44	6,925	315	1,391	152	3.0 (2.4–3.6)	2.5 (2.0–3.2)
PE Women age 45–54	8,482	532	1,696	223	3.0 (2.6–3.6)	2.5 (2.0–3.0)
PE Women age 55–66	13,747	493	2,749	202	2.3 (2.8–4.0)	2.8 (2.3–3.4)
**DVT**						
DVT Men overall	78,125	3,004	15,625	1,583	3.1 (3.0–3.4)	2.4 (2.2–2.6)
DVT Men age 25–34	6,729	220	1,345	193	5.3 (4.3–6.5)	3.2 (2.5–4.2)
DVT Men age 35–44	14,029	640	2,799	428	4.4 (3.9–5.0)	3.1 (2.6–3.6)
DVT Men age 45–54	23,008	1,284	4,601	603	2.7 (2.5–3.0)	2.2 (1.9–2.4)
DVT Men age 55–66	34,359	860	6,880	359	2.5 (2.2–2.8)	1.9 (1.7–2.3)
DVT Women overall	65,690	3,397	13,138	1,481	2.6 (2.4–2.7)	2.0 (1.8–2.1)
DVT Women age 25–34	12,416	377	2,473	229	3.4 (2.9–4.0)	2.5 (2.0–3.0)
DVT Women age 35–44	15,503	849	3,098	388	2.7 (2.4–3.0)	2.0 (1.7–2.3)
DVT Women age 45–54	17,442	1,268	3,509	524	2.4 (2.2–2.7)	1.8 (1.6–2.1)
DVT Women age 55–66	20,329	903	4,058	340	2.3 (2.0–2.7)	1.9 (1.6–2.2)

Model 1: unadjusted model controlled for matching variables by study design.

Model 3: adjusted for SES score (education, employment status, and income), obesity, cancer, coronary heart disease (including atrial fibrillation), diabetes, stroke, COPD, acute kidney failure and chronic kidney disease, surgery 3 months prior to the VTE/index date, and Charlson Comorbidity Index score, excluding comorbidities already adjusted for.

CI, confidence interval; COPD, chronic obstructive pulmonary disease; DP, disability pension; DVT, deep vein thrombosis; HR, hazard ratio; IR, incidence rate; PE, pulmonary embolism; SES, socioeconomic status; VTE, venous thromboembolism.

The risk estimates for receipt of a disability pension were essentially similar for unprovoked (HR 2.3, 95% CI: 2.2 to 2.4) and provoked VTE (HR 2.4, 95% CI: 2.0 to 2.8) (Table 3). The association between provoked VTE and receipt of a disability pension gradually increased with age for women, while it decreased with age for men. The association between unprovoked VTE and receipt of a disability pension declined with age for both sexes (Table 6).

**Table 6:** Subgroup analysis by age and sex with IRs and HRs with 95% CIs of receipt of a work-related DP among persons with and without VTE by provoked VTE vs. unprovoked VTE.

	Comparison cohort		VTE		Model 1	Model 3
	No DP	DP	No DP	DP	HR (95% CI)	HR (95% CI)
**Unprovoked VTE**						
Overall	163,940	7,044	32,788	3,354	2.8 (2.7–2.9)	2.3 (2.2–2.4)
Age 25–34	21,183	621	4,225	427	4.0 (3.5–4.6)	2.6 (2.2–3.0)
Age 35–44	33,800	1,617	6,756	890	3.3 (3.0–3.6)	2.5 (2.3–2.8)
Age 45–54	46,213	2,792	9,249	1,236	2.5 (2.4–2.7)	2.1 (2.0–2.3)
Age 55–66	62,744	2,014	12,558	801	2.4 (2.2–2.6)	2.2 (2.0–2.4)
**Provoked VTE**						
Overall	54,905	2,193	10,981	1,061	3.6 (3.3–3.9)	2.4 (2.0–2.8)
Age 25–34	5,889	204	1,168	123	3.5 (2.8–4.4)	3.0 (1.9–4.6)
Age 35–44	8,246	432	1,656	201	3.1 (2.6–3.7)	2.2 (1.5–3.2)
Age 45–54	13,552	818	2,713	387	3.6 (3.2–4.1)	2.2 (1.7–2.9)
Age 55–66	27,218	739	5,444	350	4.0 (3.5–4.6)	2.4 (1.9–3.2)

Model 1: unadjusted model controlled for matching variables by study design.

Model 3: adjusted for SES score (education, employment status, and income), obesity, cancer, coronary heart disease (including atrial fibrillation), diabetes, stroke, COPD, acute kidney failure and chronic kidney disease, surgery 3 months prior to the VTE/index date, and Charlson Comorbidity Index score, excluding comorbidities already adjusted for.

CI, confidence interval; COPD, chronic obstructive pulmonary disease; DP, disability pension; HR, hazard ratio; IR, incidence rate; SES, socioeconomic status; VTE, venous thromboembolism.

**Fig 1:**
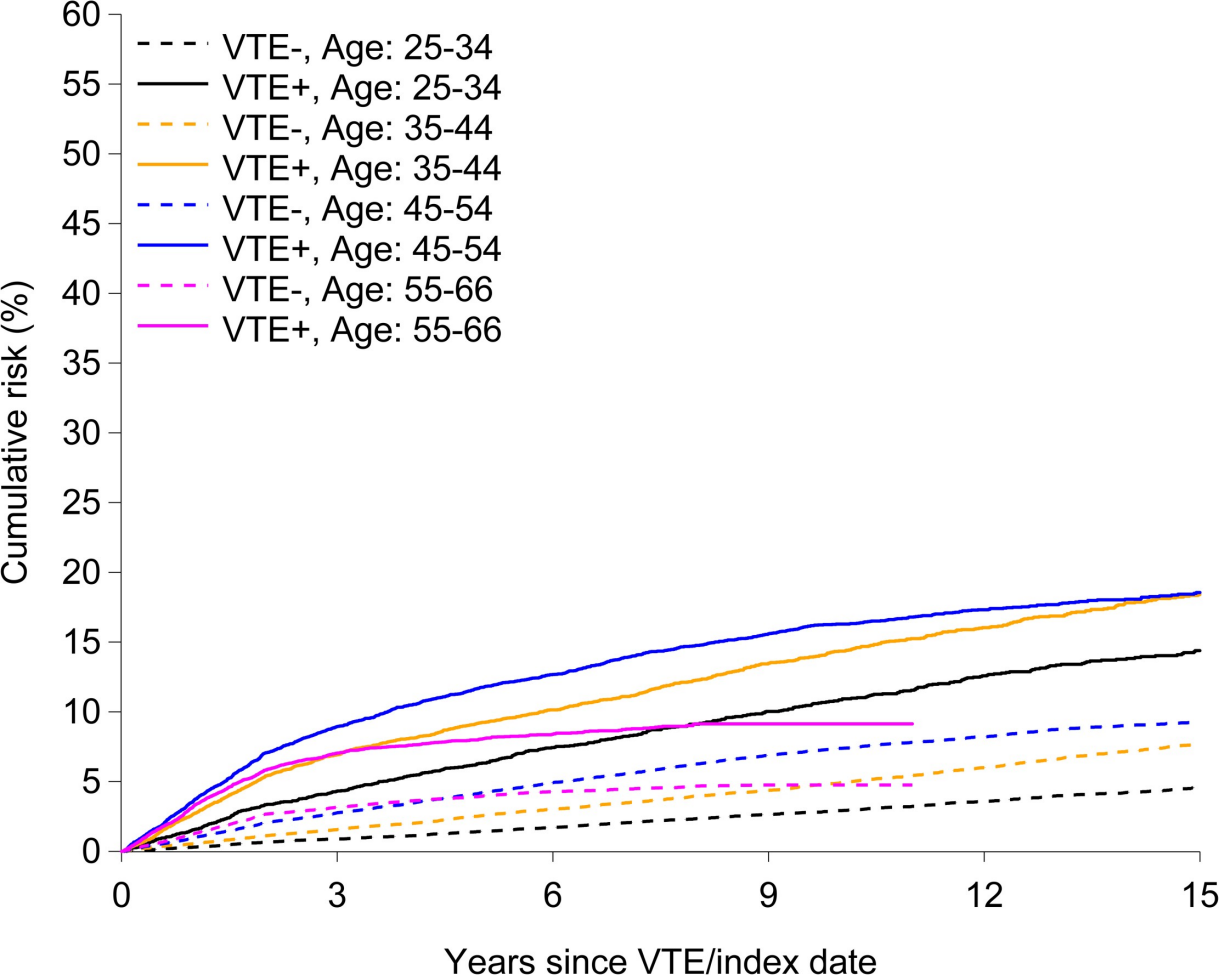
Cumulative incidence of persons with (VTE+) and without (VTE−) venous thromboembolism (VTE) receiving a work-related disability pension taking competing risk death into account.

Cumulative incidence functions (Fig 1) showed an association between VTE and disability pension in all age groups, even after competing risk by death was taken into account. The cumulative incidence of a disability pension associated with VTE in the 2 middle age groups (age 45 to 54 and 35 to 44 years) was 11.8% (95% CI, 11.1% to 12.4%) and 9.2% (95% CI, 8.6% to 9.9%), respectively, after 5 years, when death was considered as a competing event.

## Discussion

We found that VTE was associated with a 2- to 3-fold higher risk of subsequently receiving a permanent disability pension. The relative risk of disability pension receipt after VTE was highest among the youngest patients, and PE was associated with an overall higher relative risk than DVT. Unlike the general population, VTE was associated with a higher risk of receiving a disability pension in men than in women. Although the association between VTE and disability pension receipt was attenuated by adjustments for comorbidities and competing risk of death, the association was still significantly increased, thus suggesting that the relationship was not explained by the presence diseases adjusted for, or by an increased mortality in individuals with VTE. The magnitude of our risk estimates for receipt of a disability pension after VTE was essentially similar to findings from a Finnish cohort study investigating the risk of work-related disability pension following cerebrovascular and heart diseases [33]. As incident VTE is a prevalent disease also at working ages, this has the potential to cause a substantial impact on the socioeconomic challenges of VTE in the society.

The association with disability pension was higher for PE than for DVT. In contrast to this higher risk in PE, the Norwegian study [14] reported a 53% increased risk of receiving a disability pension in DVT, while no association was found between PE and disability. In the 2 European studies, the proportion who returned to work after 1 year was marginally lower in individuals with DVT compared to individuals with PE [18,19]. The PTS, characterized by pain, swelling, and reduced mobility of the affected limb, occurs in 20% to 50% of individuals with DVT [7] and may explain the increased risk of work-related disability after DVT. More recent studies investigating long-term complications of PE have reported that the post-PE syndrome affects almost 50% of individuals with PE, with symptoms and signs ranging from persistent dyspnea to life-threating chronic thromboembolic pulmonary hypertension (CTEPH) [10–13,34–39]. The post-PE syndrome reduces QoL and health-related quality of life (HRQoL) in individuals with PE [12,34–38], and CTEPH in particular is associated with poor QoL and reduced exercise capacity [37,39]. Low self-rated QoL and HRQoL have been associated strongly with subsequent health outcomes, such as sick leave [40–42]. Thus, the frequency of the post-PE syndrome, and its impact on physical function and QoL, may explain why individuals with PE suffer an equal or higher risk of work-related disability compared to individuals with DVT.

Data from studies in countries with universal welfare schemes and high female work participation have consistently shown that women overall are at higher risk of sick leave and disability pension than men [43–46]. Self-perceived health, family situation, work factors, and educational level have been proposed to explain this sex difference [43–46]. In our general comparison cohort, we found that the IR for receipt of a disability pension among women was equal to, or higher, than that for men. In contrast, the overall risk of receiving a disability pension after VTE was higher in men than women, and this sex difference in risk was driven by a higher risk in the youngest men. A higher risk of recurrence, PTS, and the post-PE syndrome in men compared to women could potentially explain this observation [6,47,48].

Our study has several strengths and limitations. The study was conducted in a setting where educational and healthcare services are government funded and free of charge to all citizens, thus preventing selection and referral bias. We used a large population-based cohort, consisting of the entire Danish population, with a long follow-up time. A validation study of DNPR data reported positive predictive values of 86% for DVT and 90% for PE, suggesting a low misclassification rate of VTE exposure [49]. Our outcome measure of work-related disability pension originated from highly accurate official registries. The large number of individuals with VTE allowed for analyses stratified on age and sex and a detailed interpretation of the association between VTE and disability. However, as disability schemes vary, our results might not be directly generalizable to other countries of time periods. We were only able to include information that was available in medical and administrative registers. We therefore lacked information on the quality and outcome of post-VTE care, individual health behavior, and workplace factors, which all are linked to receipt of a disability pension in the general population. Further, we had no information about the incidence of the post-PE syndrome, the PTS, or recurrence during follow-up in our individuals with VTE. Thus, even though we adjusted for multiple comorbidities and socioeconomic factors, residual confounding due to unmeasured factors cannot be completely ruled out.

As VTE is a prevalent disease also at working ages, our findings indicate that indirect costs due to loss of working ability may contribute substantially to the socioeconomic challenges of VTE in the society. The impact of the post-thrombotic and post-PE syndromes as mediators for permanent work-related disability is not well studied, and future research should focus on identifying determinants and risk factors for permanent loss of working ability in individuals with VTE.

In conclusion, VTE was associated with future risk of permanent work-related disability, and the association was still observed after accounting for comorbidities such as cancer and other cardiovascular diseases. Our results emphasize the social consequences of VTE and can help occupational and healthcare professionals to identify vulnerable individuals at risk of permanent exclusion from the labor market after a VTE event.

## Supporting information

S1 STROBE Checklist STROBE guideline. (PDF)

S1 Analysis plan Prospective analysis plan. (PDF)

S1 Fig Restricted cubic spline model with adjusted HRs and 95% CIs of receipt of a work-related disability pension according to age at VTE diagnosis. CI, confidence interval; HR, hazard ratio; VTE, venous thromboembolism.
(TIF)

S1 Table Baseline characteristics of persons with VTE and members of the general comparison cohort. VTE, venous thromboembolism.
(TIF)

S2 Table Characteristics of patients with VTE with and without work-related disability pension. VTE, venous thromboembolism.
(TIF)

S3 Table International Standard Classification of Education − 2011 English and Danish educational program names and corresponding levels of education. (TIF)

S4 Table Division of level of education (Danish levels) based on S3 Table. (TIF)

S5 Table ICD and ATC classification codes used to define exposure, provoking factors for VTE, covariables, and modified Charlson Comorbidity Index. ATC, Anatomical Therapeutic Chemical Classification; ICD, International Classification of Diseases; VTE, venous thromboembolism.
(TIF)

## Abbreviations

CI: confidence interval
COPD: chronic obstructive pulmonary disease
CRS: Civil Registration System
CTEPH: chronic thromboembolic pulmonary hypertension
DNPR: Danish National Patient Registry
DVT: deep vein thrombosis
HR: hazard ratio
HRQoL: health-related quality of life
IDA: Integrated Database for Labour Market Research
IR: incidence rate
PE: pulmonary embolism
post-PE syndrome: post-pulmonary embolism syndrome
PTS: post-thrombotic syndrome
QoL: quality of life
SES: socioeconomic status
VTE: venous thromboembolism
