# Supplementary material for: Risk of a permanent work-related disability pension after incident venous thromboembolism in Denmark: A population-based cohort study
Source: PLoS Med. 2021 Aug 31;18(8):e1003770. doi: 10.1371/journal.pmed.1003770 (PMC8443033; doi:10.1371/journal.pmed.1003770)
Supplement: S1 STROBE Checklist — (PDF) [file pmed.1003770.s001.pdf]

S1 STROBE Checklist - **Risk of a permanent work-related disability pension after incident venous thromboembolism in Denmark: A population-based cohort study**

	Item No	Recommendation	Paragraph in manuscript
**Title and abstract**	1	(*a*) Indicate the study's design with a commonly used term in the title or the abstract	In Methods and findings, paragraph 1
		(*b*) Provide in the abstract an informative and balanced summary of what was done and what was found	In Methods and findings, paragraph 1 and 2
**Introduction**			
Background/rationale	2	Explain the scientific background and rationale for the investigation being reported	In Introduction paragraph 1 and 2
Objectives	3	State specific objectives, including any prespecified hypotheses	In Introduction paragraph 2
**Methods**			
Study design	4	Present key elements of study design early in the paper	In Design and setting paragraph 1
Setting	5	Describe the setting, locations, and relevant dates, including periods of recruitment, exposure, follow-up, and data collection	In VTE cohort, paragraph 1
Participants	6	(*a*) Give the eligibility criteria, and the sources and methods of selection of participants. Describe methods of follow-up	In VTE cohort, paragraph 1 and 2, General comparison cohort, paragraph 1
		(*b*) For matched studies, give matching criteria and number of exposed and unexposed	In VTE cohort, paragraph 1 and 2, General comparison cohort, paragraph 1
Variables	7	Clearly define all outcomes, exposures, predictors, potential confounders, and effect modifiers. Give diagnostic criteria, if applicable	In VTE cohort, paragraph 1 and 2, General comparison cohort, paragraph 1
Data sources/ measurement	8*	For each variable of interest, give sources of data and details of methods of assessment (measurement). Describe comparability of assessment methods if there is more than one group	In VTE cohort, paragraph 1 and 2, General comparison cohort, outcome paragraph 1 and 2, and cohort statistics paragraph 1 and 2
Bias	9	Describe any efforts to address potential sources of bias	In VTE cohort paragraph 1 and 2, Outcome paragraph 2, cohort characteristics paragraph 2
Study size	10	Explain how the study size was arrived at	In VTE cohort paragraph 1 and general comparison cohort paragraph 1
Quantitative variables	11	Explain how quantitative variables were handled in the analyses. If applicable, describe which groupings were chosen and why	In cohort characteristics paragraph 1-3

Statistical methods	12	(*a*) Describe all statistical methods, including those used to control for confounding	In statistical analysis paragraph 1-3
		(*b*) Describe any methods used to examine subgroups and interactions	In statistical analysis paragraph 2-3
		(*c*) Explain how missing data were addressed	N/A
		(*d*) If applicable, explain how loss to follow-up was addressed	
		(*e*) Describe any sensitivity analyses	In statistical analysis paragraph 2-3
**Results**			
Participants	13*	(a) Report numbers of individuals at each stage of study—eg numbers potentially eligible, examined for eligibility, confirmed eligible, included in the study, completing follow-up, and analysed	In results paragraph 1 and 2, and tables 1, 2 and supplementary table 1
		(b) Give reasons for non-participation at each stage	N/A
		(c) Consider use of a flow diagram	N/A
Descriptive data	14*	(a) Give characteristics of study participants (eg demographic, clinical, social) and information on exposures and potential confounders	In results paragraph 1 and 2, and tables 1, 2 and supplementary table 1
		(b) Indicate number of participants with missing data for each variable of interest	N/A
		(c) Summarise follow-up time (eg, average and total amount)	In results paragraph 2
Outcome data	15*	Report numbers of outcome events or summary measures over time	In results paragraph 2
Main results	16	(*a*) Give unadjusted estimates and, if applicable, confounder-adjusted estimates and their precision (eg, 95% confidence interval). Make clear which confounders were adjusted for and why they were included	In results paragraph 3 and table 2
		(*b*) Report category boundaries when continuous variables were categorized	N/A
		(*c*) If relevant, consider translating estimates of relative risk into absolute risk for a meaningful time period	In results paragraph 3
Other analyses	17	Report other analyses done—eg analyses of subgroups and interactions, and sensitivity analyses	In results paragraph 4-9 and tables 3-5.
**Discussion**			
Key results	18	Summarise key results with reference to study objectives	In discussion paragraph 1

Limitations	19	Discuss limitations of the study, taking into account sources of potential bias or imprecision. Discuss both direction and magnitude of any potential bias	In discussion paragraph 4
Interpretation	20	Give a cautious overall interpretation of results considering objectives, limitations, multiplicity of analyses, results from similar studies, and other relevant evidence	In discussion paragraph 2-3
Generalisability	21	Discuss the generalisability (external validity) of	In discussion paragraph 4
		the study results	
**Other information**			
Funding	22	Give the source of funding and the role of the funders for the present study and, if applicable, for the original study on which the present article is based	N/A

*Give information separately for exposed and unexposed groups.
