# Supplementary material for: Risk of a permanent work-related disability pension after incident venous thromboembolism in Denmark: A population-based cohort study
Source: PLoS Med. 2021 Aug 31;18(8):e1003770. doi: 10.1371/journal.pmed.1003770 (PMC8443033; doi:10.1371/journal.pmed.1003770)
Supplement: S1 Analysis plan — (PDF) [file pmed.1003770.s002.pdf]

Analysis Plan

Title:

Risk of a permanent work-related disability pension after incident venous thromboembolism

Project group:

Helle Jørgensen, John-Bjarne Hansen, Sigrid Brækkan, Erzsébet Horváth-Puhó, Kristina Laugesen, Henrik Toft Sørensen

Objective:

To assess the risk of receiving a permanent work-related disability pension among patients with VTE compared to individuals without VTE and assess whether this association was explained by comorbidities such as cancer and arterial cardiovascular disease.

Background and hypothesis:

Venous thromboembolism (VTE), encompassing deep vein thrombosis (DVT) and pulmonary embolism (PE), is a prevalent chronic disease.[1,2] Despite increased awareness and availability of preventive measures, VTE incidence has increased during the past decades.[3,4] Although VTE has been documented as a leading cause of lost disability-adjusted life-years, existing research on work-related disability and socioeconomic consequences following a VTE is scarce.[6,5] Two European multicenter studies found that 27.8% of the PE patients and 29.5% of the DVT patients had not returned to work one year after their VTE diagnosis.[6,7]A Norwegian cohort study reported a 37% increased risk of permanent work-related disability in VTE patients compared to the general population, with a higher risk in DVT patients than in PE patients.[8] Thus, VTE represents a major burden to public health and health care systems.[5,9,10] Further research on the work-related consequences of VTE is required to improve protective strategies that diminish the indirect costs and social burden of the disease.

Methods:

Overview of study design: A Danish nationwide population-based cohort study consisting of individuals aged 25-66 years with incident VTE during 1995-2016 and a comparison cohort from the general population matched on birth year, sex, and calendar-year.

Study subjects: Individuals aged 25-66 years with in- or outpatient VTE diagnosis in the period 1995-2016, For each VTE patients we will frequency match five individuals (by birth year, sex and calendar year) from the general population, with replacements.

Data sources: Demographic information and data on vital status and migration will be extracted from the Danish Civil Registration System (CRS). Information on VTE and comorbidities will be obtained from the Danish National Patient Registry (DNPR). We will obtain data on disability pension, income and employment status from the Integrated Database for Labor Market Research, and data on education from the Educational Attainment Register.

Selection criteria for the study population: We will use data from the DPNR (ICD-10 and -8 codes) to identify all inpatients and outpatients with a first-lifetime primary or secondary diagnosis of DVT, PE, splanchnic vein thrombosis or cerebral vein thrombosis in the period January 1, 1995 through December 31, 2016.

For each VTE patient up to five individuals from the general population will be randomly sampled matched on sex, year of birth, and calendar year, with replacement. The VTE diagnosis date for the corresponding VTE patient will be defined as the index date for the comparison cohort member. Study

participants cannot have been hospitalized for VTE or have received disability pension prior to the index date. If a person from the comparison cohort subsequently experiences a VTE, he or she will be censored and moved to the appropriate exposure cohort from that date onwards.

Measurements:

Main exposure variable: In- or outpatient VTE

Potential confounding variables: Age, sex, body mass index, socioeconomic status (combined measure of education, income and employment status), and comorbidities

Outcome variables: Permanent disability pension

Statistical issues

Person-time of follow-up will be accrued from the index date until the 1st of January the year the disability pension recorded (i.e. follow-up stops 11 months before DP is recorded), emigration from Denmark, date of death or end of the study period (Dec 31, 2016), whichever comes first. Cox proportional hazard regression models will be used to estimate hazard ratios with 95% confidence interval for the various exposures. Additionally, age-stratified analyses (age groups: 25, 40 and 55 on the date of inclusion) will be performed. Patients aged <25 and >66 years will be excludes as they are unlikely to be eligible for the outcome. In order to account for death as a competing event, cumulative incidence functions will also be estimated.

References

1. White RH. The epidemiology of venous thromboembolism. *Circulation* 2003; **107**(23 Suppl 1): I4-8.
2. Heit JA, Spencer FA, White RH. The epidemiology of venous thromboembolism. *J Thromb Thrombolysis* 2016; **41**(1): 3-14.
3. Huang W, Goldberg RJ, Anderson FA, Kiefe CI, Spencer FA. Secular trends in occurrence of acute venous thromboembolism: the Worcester VTE study (1985-2009). *Am J Med* 2014; **127**(9): 829-39 e5.
4. Arshad N, Isaksen T, Hansen JB, Braekkan SK. Time trends in incidence rates of venous thromboembolism in a large cohort recruited from the general population. *Eur J Epidemiol* 2017; **32**(4): 299-305.
5. Grosse SD, Nelson RE, Nyarko KA, Richardson LC, Raskob GE. The economic burden of incident venous thromboembolism in the United States: A review of estimated attributable healthcare costs. *Thromb Res* 2016; **137**: 3-10.
6. Willich SN, Chuang LH, van Hout B, et al. Pulmonary embolism in Europe - Burden of illness in relationship to healthcare resource utilization and return to work. *Thromb Res* 2018; **170**: 181-91.
7. Chuang LH, van Hout B, Cohen AT, et al. Deep-vein thrombosis in Europe - Burden of illness in relationship to healthcare resource utilization and return to work. *Thromb Res* 2018; **170**: 165-74.
8. Braekkan SK, Grosse SD, Okoroh EM, et al. Venous thromboembolism and subsequent permanent work-related disability. *Journal of thrombosis and haemostasis : JTH* 2016; **14**(10): 1978-87.
9. Cohen AT, Agnelli G, Anderson FA, et al. Venous thromboembolism (VTE) in Europe. The number of VTE events and associated morbidity and mortality. *Thromb Haemost* 2007; **98**(4): 756-64.
10. Raskob GE, Angchaisuksiri P, Blanco AN, et al. Thrombosis: a major contributor to global disease burden. *Arterioscler Thromb Vasc Biol* 2014; **34**(11): 2363-71.

Tables

ICD- and ATC codes to define exposure, provoking factors for VTE, covariables and Modified Charlson Comorbidity Index

Variables	ICD-8 codes	ICD-10 codes	ATC codes
**Exposure**			
Pulmonary embolism	45099	I26	
Deep venous thrombosis	45100	I801; I802; I803	
**Provoking factors for VTE**			
Fracture	800-929; 950-959	S00-T14	
Surgery	00000-99960	KA-KQ; KX; KY	
Pregnancy	630-680	O00-O99	
Cancer	140-209 (except 172)	C00- C97 (except C44)	
**Covariables**			
Coronary heart disease (including myocardial infarction and atrial fibrillation)	41009; 41099; 41109; 41199; 41209; 41299; 41309; 41399; 41409; 41499; 42793; 42794	I20-I25; I48	
Diabetes mellitus type I or II	249; 250	E10; E11; E14	A10
Chronic obstructive pulmonary disease (COPD)	490–493	J40-J49	
Obesity	277.99	E65-E68	
Stroke (hemorrhagic or ischemic)	430; 431; 432; 433; 434; 435; 437.0; 437.1	I60; I61;I62; I63; I65; I66; I67.2; I67.8	
Acute kidney failure and chronic kidney disease	403; 404; 580-584; 590.09; 593.19; 753.10-753.19; 792	I12; I13; N00-N05; N07; N11; N14; N17- N19; Q61	
**Modified Charlson Comorbidity Index**			
Congestive heart failure	427.09; 427.10; 427.11; 427.19; 428.99; 782.49	I50; I11.0; I13.0; I13.2	
Peripheral vascular disease	440; 441; 442; 443; 444; 445	I70; I71; I72; I73; I74; I77	
Cerebrovascular disease	438	I68-I69; G45; G46	
Dementia	290.09-290.19; 293.09	F00-F03; F05.1; G30	
Chronic pulmonary disease	515-518	J60-J67; J68.4; J70.1; J70.3; J84.1; J92.0; J96.1; J98.2; J98.3	
Connective tissue disease	712; 716; 734; 446; 135.99	M05; M06; M08; M09;M30;M31; M32; M33; M34; M35; M36; D86	
Ulcer disease	530.91; 530.98; 531-534	K22.1; K25-K28	
Mild liver disease	571; 573.01; 573.04	B18; K70.0-K70.3; K70.9; K71; K73; K74; K76.0;	
Hemiplegia	344	G81; G82	
Moderate to severe liver disease	070.00; 070.02; 070.04; 070.06; 070.08; 573.00; 456.00-456.09	B15.0; B16.0; B16.2; B19.0; K70.4; K72; K76.6; I85	
AIDS	079.83	B21-B24	

Table 1 Characteristics of VTE cohort and matched comparison cohort by age groups

	Age 25-34				Age 35-44				Age 45-54				Age 55-66				Overall			
	VTE cohort		Comparison cohort		VTE cohort		Comparison cohort		VTE cohort		Comparison cohort		VTE cohort		Comparison cohort		VTE cohort		Comparison cohort	
	N	%	N	%	N	%	N	%	N	%	N	%	N	%	N	%	N	%	N	%
Total																				
Gender																				
Male																				
Female																				
Year of VTE diagnosis																				
1995-1999																				
2000-2004																				
2005-2009																				
2010-2016																				
Personal annual income the year before index date																				
Low																				
Middle																				
Medium high																				
High																				
Missing																				

**Primary employment status the year before index date**																				
**Unemployed**																				
**Outside workforce**																				
**Employed**																				
**Missing**																				
**Level of education the year before index date**																				
**Low**																				
**Middle**																				
**High**																				
**Missing**																				
**SES score**																				
**Low**																				
**Medium**																				
**High**																				
**Missing**																				
**Cancer**																				
**No**																				
**Yes**																				
**Coronary heart disease**																				
**No**																				
**Yes**																				
**Diabetes**																				
**No**																				
**Yes**																				

**COPD**																				
**No**																				
**Yes**																				
**Obesity**																				
**No**																				
**Yes**																				
**Stroke**																				
**No**																				
**Yes**																				
**Charlson com. Index**																				
**No**																				
**Yes**																				
**Acute kidney failure and chronic kidney disease**																				
**No**																				
**Yes**																				
**Surgery 3 months prior to VTE**																				
**No**																				
**Yes**																				

Table 2 Characteristics of patients with venous thromboembolism (VTE) with and without work-related disability

	Age 25-34				Age 35-44				Age 45-54				Age 55-66				Overall			
	No work-related disability after VTE		Work-related disability after VTE		No work-related disability after VTE		Work-related disability after VTE		No work-related disability after VTE		Work-related disability after VTE		No work-related disability after VTE		Work-related disability after VTE		No work-related disability after VTE		Work-related disability after VTE	
	N	%	N	%	N	%	N	%	N	%	N	%	N	%	N	%	N	%	N	%
**Total**																				
**Male**																				
**Female**																				
**Pulmonary Embolism**																				
**Deep Vein Thrombosis**																				
**Unprovoked VTE**																				
**Provoked**																				

Table 3 Incidence rate and hazard ratios with 95 % confidence intervals of work-related disability pension after VTE

Category	Age	CC: No. at Risk	CC: Events	CC: IR	VTE: No. at Risk	VTE: Events	VTE: IR	Model 1 HR (95 % CI)	Model 2 HR (95 % CI)	Model 3 HR (95 % CI)	Model 4 HR (95 % CI)
VTE	Overall										
VTE	25-34										
VTE	34-44										
VTE	45-54										
VTE	55-66										
VTE - M	Overall										
VTE - M	25-34										
VTE - M	34-44										
VTE - M	45-54										
VTE - M	55-66										
VTE - F	Overall										
VTE - F	25-34										
VTE - F	34-44										
VTE - F	45-54										
VTE - F	55-66										

Model 1:Unadjusted model controlled for matching variables by study design
Model 2: Adjusted for socioeconomic status (education, employment status and income) and obesity
Model 3: Adjusted for Adjusted for socioeconomic status (education, employment status and income), obesity, cancer, Coronary Heart Disease (including atrial fibrillation), diabetes, stroke, COPD, Acute kidney failure and chronic kidney disease, Surgery 3 months prior to VTE
Model 4: Adjusted for Adjusted for socioeconomic status (education, employment status and income), obesity, cancer, Coronary Heart Disease (including atrial fibrillation), diabetes, stroke, COPD, Acute kidney failure and chronic kidney disease, Surgery 3 months prior to VTE, Charlson comorbidity index excluding comorbidities already adjusted for in model 3

Table 4 Subgroup analysis for incidence rate and hazard ratios with 95 % confidence intervals of work-related disability pension after VTE

Category	Age	CC: No. at Risk	CC: Events	CC: IR	VTE: No. at Risk	VTE: Events	VTE: IR	Model 1 HR (95 % CI)	Model 2 HR (95 % CI)	Model 3 HR (95 % CI)	Model 4 HR (95 % CI)
DVT	Overall										
DVT	25-34										
DVT	34-44										
DVT	45-54										
DVT	55-66										
DVT-M	Overall										
DVT-M	25-34										
DVT-M	34-44										
DVT-M	45-54										
DVT-M	55-66										
DVT-F	Overall										
DVT-F	25-34										
DVT-F	34-44										
DVT-F	45-54										
DVT-F	55-66										
PE	Overall										
PE	25-34										
PE	34-44										
PE	45-54										
PE	55-66										
PE-M	Overall										

PE-M	25-34										
PE-M	34-44										
PE-M	45-54										
PE-M	55-66										
PE-F	Overall										
PE-F	25-34										
PE-F	34-44										
PE-F	45-54										
PE-F	55-66										
Unprovoked	Overall										
Unprovoked	25-34										
Unprovoked	34-44										
Unprovoked	45-54										
Unprovoked	55-66										
Unprovoked-M	Overall										
Unprovoked-M	25-34										
Unprovoked-M	34-44										
Unprovoked-M	45-54										
Unprovoked-M	55-66										
Unprovoked-F	Overall										
Unprovoked-F	25-34										
Unprovoked-F	34-44										

Unprovoked-F	45-54										
Unprovoked-F	55-66										
Provoked	Overall										
Provoked	25-34										
Provoked	34-44										
Provoked	45-54										
Provoked	55-66										
Provoked -M	Overall										
Provoked -M	25-34										
Provoked -M	34-44										
Provoked -M	45-54										
Provoked -M	55-66										
Provoked -F	Overall										
Provoked -F	25-34										
Provoked -F	34-44										
Provoked -F	45-54										
Provoked -F	55-66										

Model 1: Unadjusted model controlled for matching variables by study design
Model 2: Adjusted for socioeconomic status (education, employment status and income) and obesity
Model 3: Adjusted for socioeconomic status (education, employment status and income), obesity, cancer, Coronary Heart Disease (including atrial fibrillation), diabetes, stroke, COPD, Acute kidney failure and chronic kidney disease, Surgery 3 months prior to VTE
Model 4: Adjusted for socioeconomic status (education, employment status and income), obesity, cancer, Coronary Heart Disease (including atrial fibrillation), diabetes, stroke, COPD, Acute kidney failure and chronic kidney disease, Surgery 3 months prior to VTE, Charlson comorbidity index excluding comorbidities already adjusted for in model 3
