# Supplementary figures and images for: Risk of a permanent work-related disability pension after incident venous thromboembolism in Denmark: A population-based cohort study
Source: PLoS Med. 2021 Aug 31;18(8):e1003770. doi: 10.1371/journal.pmed.1003770 (PMC8443033; doi:10.1371/journal.pmed.1003770)

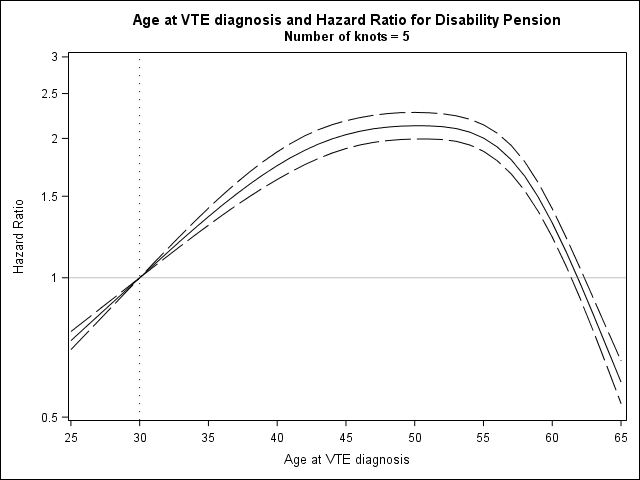

Supplement: S1 Fig — CI, confidence interval; HR, hazard ratio; VTE, venous thromboembolism. (TIF) [file pmed.1003770.s003.tif]

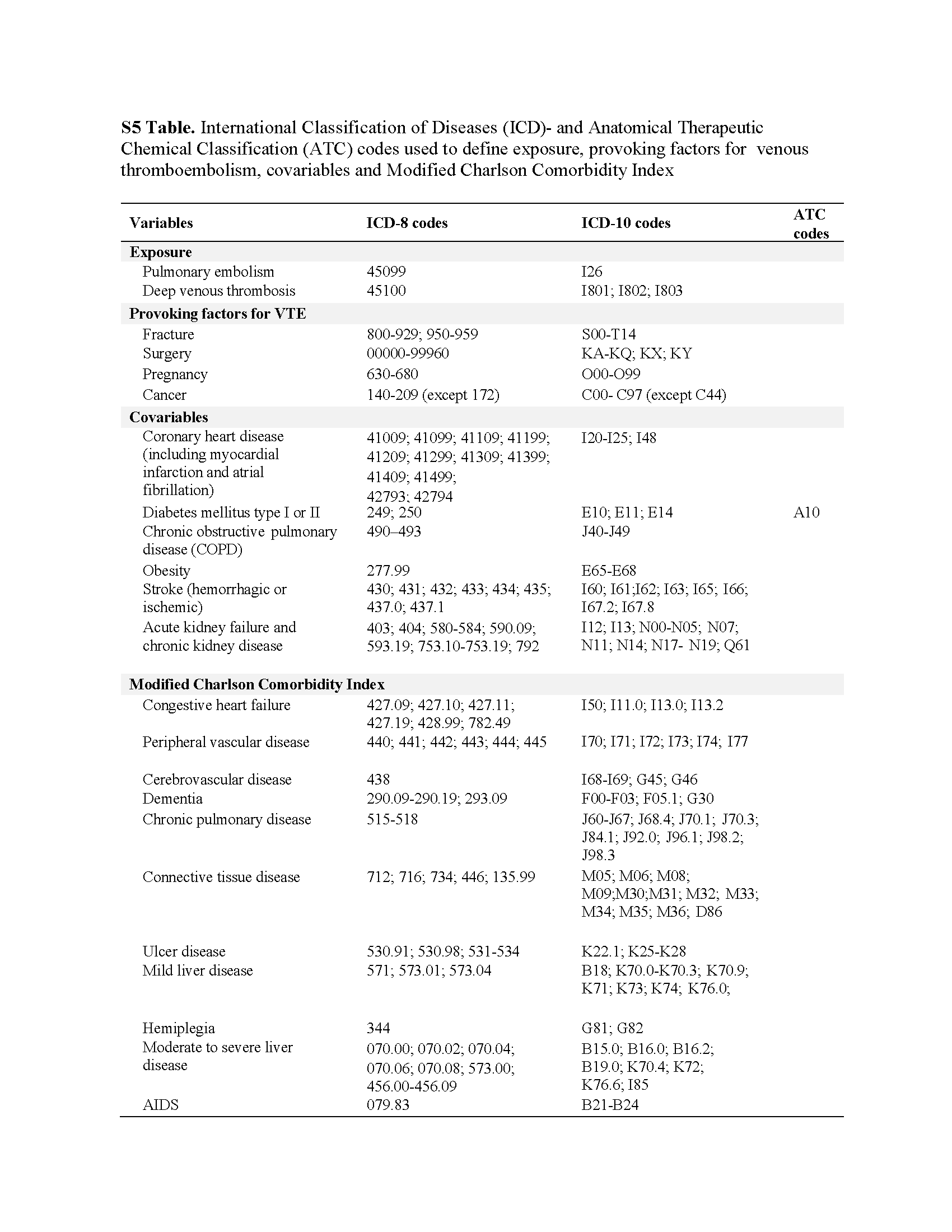

Supplement: S5 Table — ATC, Anatomical Therapeutic Chemical Classification; ICD, International Classification of Diseases; VTE, venous thromboembolism. (TIF) [file pmed.1003770.s008.tif]
